# Financial Expenses and “Losses” of the Polish Healthcare System Resulting from the Occurrence of Adverse Events

**DOI:** 10.3390/ijerph19137932

**Published:** 2022-06-28

**Authors:** Tomasz Leśniak, Aleksandra Sierocka, Dariusz Kostrzewa, Remigiusz Kozłowski, Michał Marczak

**Affiliations:** 1 Lesta Law Firm, 45-316 Opole, Poland; t.lesniak@kancelarialesta.pl; 2 Department of Management and Logistics in Healthcare, Medical University of Lodz, 90-419 Lodz, Poland; michal.marczak@umed.lodz.pl; 3 COPERNICUS Medical Entity, 80-803 Gdansk, Poland; dariusz.kostrzewa@wss.gda.pl; 4 Centre for Security Technologies in Logistics, Faculty of Management, University of Lodz, 90-237 Lodz, Poland; remigiusz.kozlowski@wz.uni.lodz.pl

**Keywords:** risk management, adverse events, secondary harm, cost of treatment, damages

## Abstract

Background: The globally increasing healthcare expenditures related to the need to treat the consequences of adverse events, as well as the number of claims filed by patients (or their families) and remuneration paid as their result mean that the interest in the subject of adverse event cost management is increasing. An increase in the number of cases concerning medical errors has also occurred in Poland in recent years. The newest statistics from the Ministry of Justice demonstrate that the courts are awarding increasingly higher amounts. The goal of this work was an attempt to approximate, based on our own experiences, the impact of adverse events on the expenditures of the healthcare system in Poland, including the costs of treatment of the consequences of such events, described by the authors as “secondary harm”. Methods: Based on the analysis of 100 cases for compensation for the occurrence of a medical event, an initial estimate of the costs of primary (initial) treatment, which resulted in the occurrence of the adverse event, and the costs of subsequent hospitalisations/stays, which were its consequences. The study was conducted in the period from October 2020 to November of 2021. Results: The statistical analysis of the examined cases enabled establishing that in 62% they concerned women. Only 38% were events which applied to men. The highest number of cases concerned events which occurred in the last years, that is 2018 (35%), 2019 (23%), and 2017 (17%). The most frequent events included those related to incorrect diagnosis (the lack of correct diagnosis), which resulted in appropriate activities not being undertaken and a lack of appropriate treatment, e.g., lack of diagnosis of cancer, myocardial infarction, appendicitis, or fracture (26%). The next one was incorrect surgical treatment (17%)—the consequence of which was most frequently a need for repeated surgery and an incorrect conservative treatment of injuries. The obtained results demonstrate that significantly higher funds are spent by medical entities for “restorative” actions (on average EUR 1433, which attempt to mitigate against the negative consequences of incorrect decisions or actions in the original treatment (average cost of EUR 814)). Conclusions: The consequences of adverse events include not only health-related harm for the patient, but also long-term social, familial, or professional results. The authors of the article are of an opinion that all the conducted analyses and conclusions drawn from them should serve the improvement of patient safety. They also form an initial point for establishing recommendations and advice for the improvement of safety and quality of medical services and the reduction of healthcare-related costs. The authors propose covering the parties injured by an adverse event (subjected to “secondary harm”) with a unique, innovative programme of post-accident health care, “Health Reconstruction”.

## 1. Introduction

The globally increasing healthcare expenditures related to the need to treat the consequences of adverse events, as well as the number of claims filed by patients (or their families) and the remuneration paid as their result mean that the interest in the subject of adverse event cost management is increasing [1,2,3]. A problem which once was only noticed by people in charge of court proceedings is now a nightmare of people who consider the improvement of quality and effective management to be the basis of appropriate and rational administration. The amount of compensation (damages) paid may also have an impact on the financial liquidity of the healthcare entity. Therefore, it is important to learn about the root causes of the given event and to draw conclusions for the future. This enables the implementation of corrective (preventive) action, which should safeguard against the repeated occurrence of similar situations in the future and against negative repercussions [4]. It should be also remembered that the resources of the medical entity, including personnel (their qualifications, experience, competences, and physical and mental condition) and state-of-the-art working medical equipment or rooms which meet appropriate sanitary and epidemiological standards and requirements have a significant impact on the risk of occurrence of adverse events. Old, defective equipment, overworked medical personnel, and not following procedures and standards may significantly increase this risk, causing a worsening of the patient’s health, additional complications, harm to the patient’s health, and as a consequence result in claims by the patient (or by their families) [5].

An increase in the number of cases concerning medical errors has also occurred in Poland in recent years. In 2016, prosecutor’s offices have been conducting 4963 prosecutorial proceedings, that is, almost 46% more proceedings than in 2015 (3394 proceedings), and the number of commenced proceedings for this type of crime has increased by over 23% (that is, by 408 prosecutions) [6]. The newest statistics from the Ministry of Justice demonstrate that the courts are awarding increasingly higher amounts. In the years 2014–2017, the amount of compensation and damages increased by a total of 70%. In 2017, financial compensation for harm caused in the national health care system was received by 81 persons for a total amount of EUR 3.170 million (in 2014 it was less than EUR 1.904 million). In 2017, the private hospitals were also required by courts to pay EUR 13,160 in damages and over EUR 164,500 in compensation (30% more than in 2016) [7,8].

Adverse events, defined as “harm to the patient’s health caused during the diagnostic and/or treatment, not related to the natural course of the illness or the patient’s condition, and also the risk of its occurrence” [9], are being identified in every healthcare entity. Taking into account the character and specifics of healthcare entities, the aforementioned events may occur on every stage of a patient’s treatment (from the moment of admission to the hospital until the moment of discharge). Therefore, the probability of the occurrence of such an event cannot be entirely eliminated [10]. However, this does not mean that we should not do anything with this issue. All actions that would result in the effective minimisation of the risk of adverse events to the level tolerated and/or acceptable for the patient, the medical community, and for the entire society, have to be undertaken.

The occurrence of adverse events is therefore related to a series of negative effects, harmful both to the patient (and/or his family) and to the medical personnel of healthcare entities. They include primarily physical and/or mental harm, loss of trust (not only in a specific doctor/nurse/healthcare entity, but also the entire healthcare system), as well as the decreased dedication/morale of staff. The impact of adverse events on healthcare staff is so significant that such staff are often called “the second victim” [11,12,13,14,15]. Adverse events are also expensive [16,17] (e.g., they extend hospitalisations, require the performing of repeated surgeries and diagnostics, and they force the use of above-standard drugs and materials). What is important is that they are associated with additional social costs in the form of decreased productivity and decreased population health.

All the issues described and information presented above have contributed to the establishment of this work, which was intended to give an overview, based on our own experience, of the impact of adverse events on the expenditures of the health care system in Poland. There are many examples of publications concerning adverse events occurring in healthcare entities all over the world [18], although the statistics themselves are underestimated in many cases [19]. We have also an increasing amount of data on the costs of claims and on the damages paid. The presented article is intended to also raise one aspect that is important for us (beyond the harm to the patient), related to the burden placed on the public healthcare system by the expenses resulting from the treatment of the consequences of such adverse events. The authors of the study have used the term “secondary harm”, which for the purpose of this publication was defined by the authors as a medical condition which is not directly related to the original condition of the patient in contact with the healthcare system, but a secondary medical condition which has occurred after the adverse event. The secondary harm is an event, which occurred not at the patient’s fault, but through a widely understood adverse event resulting from a diagnostic error, therapeutic error, administrative error, or another fault on the part of the healthcare system, caused by the medical facility’s personnel, a specialised organisation, or by a system of private and public facilities serving the patient. The secondary harm is therefore a medical condition which can be directly connected to an event which would not have occurred without the participation of the healthcare system. It is harm with financial consequences for the entire healthcare system (the payer, the National Health Fund, bears the expenses of additional medical procedures which should not occur were the “secondary harm” not caused).

Therefore, in almost every case, the patient should be offered an individual treatment plan prepared for them, appropriate supervision over the process of their return to health, and a selection of appropriate specialists, increasing, e.g., the quality and effectiveness of the restoration of health. Patients frequently do not even know which specialist to contact with their illnesses, and the indications of specialists who do not consult with each other may be mutually exclusive. According to the authors, the main goals should therefore include:

– Coordinating and providing aid in the daily functioning of the victim and their family;
– Access to high-quality medical, psychological, financial, compensatory, legal, and infrastructural care;
– Preventing disabilities and social and professional exclusion (including professional diseases).

**Figure 1:**
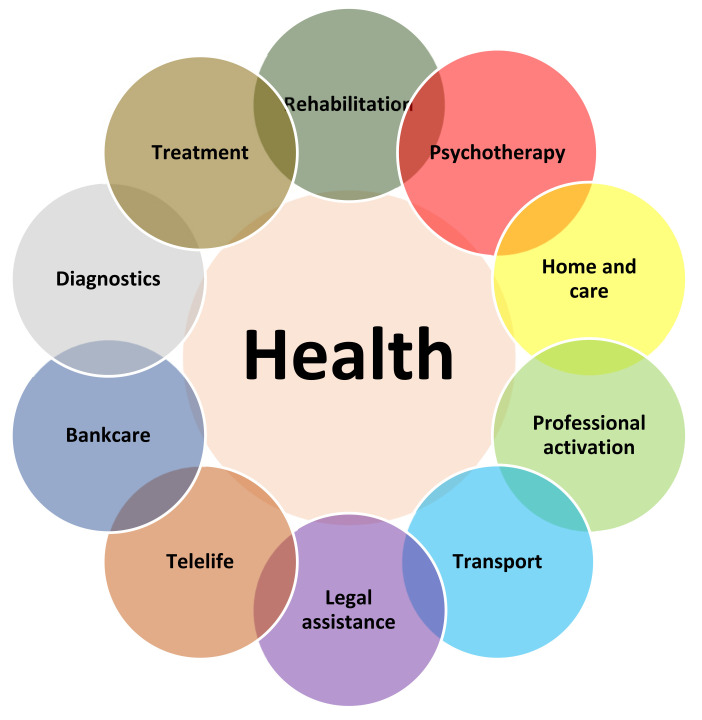
The model of health reconstruction procedure—own proposal.

The procedure could look as follows (Figure 1):

We cannot forget about such an approach and about analysing the consequences of adverse events. These are not only the closest, direct effects. These are also consequences which may last for years (even until the end of the victim’s life and/or after their death in the case of their family) which burden not only the patient, but the entire healthcare system with significant costs.

Based on the analysis of specific cases, an initial estimate is made for the costs of primary (initial) treatment, which resulted in the occurrence of the adverse event, as well as the costs of subsequent hospitalisations/stays, which were its consequences. The assessment of costs applied to the expenditure of the Polish public healthcare system that finances the treatment of patients in medical facilities which have a signed agreement with the National Health Fund (the payer). The calculation was based on National Health Fund (NFZ) directories in force in the year in question, which contained the valuation of individual services by the payer. The longer the treatment, that is, the more additional hospitalisations, studies, and thus services provided, the higher the expenses of the system and its financial burden (“secondary harm”).

## 2. Methodology

For the purpose of this study, a total of 100 cases for compensation due to an occurrence of an adverse event from the Provincial Committee Adjudicating on Medical Events of the Dolnośląskie Provincial Office in Wroclaw, from judgements of common courts and conducted by one of the law offices in the Opolskie province, were analysed. The analysis covered cases which met the following inclusion criteria:

– Assigned to one of the concepts: doctor’s error, medical error, or adverse event;
– Active or finished during the study period;
– Containing data which characterise the event (diagnosis, performed procedures, duration of hospitalization, and the type of ward, harm to patient’s health, or patient’s death);
– Containing information concerning the further treatment of the party injured as a consequence of the event/error (number and type of subsequent visits/hospitalisations);
– Containing information about own expenditures of the patient resulting from the occurrence of the event/error.

The study was conducted in the period from October 2020 to November of 2021. For every case, information was collected on:

– The patient’s age and sex;
– Hospitalisations/stays during which an adverse event has occurred, including:
  (a) Length of hospitalisations/stays (date from–to);
  (b) Primary diagnosis and co-existing diagnoses acc. to ICD-10 classification;
  (c) Applied medical procedures acc. to ICD-9 classification.
– Subsequent hospitalisations/stays which are a consequence of the adverse event (“secondary harm”), including:
  (a) Length of hospitalisations/stays (date from–to);
  (b) Primary diagnosis and co-existing diagnoses acc. to ICD-10 classification;
  (c) Applied medical procedures acc. to ICD-9 classification;
  (d) Interval between hospitalisation/stay caused by the illness, and the diagnosis of “secondary harm”;
  (e) Time of treatment as a result of the “secondary harm” (if possible to establish);
  (f) Remaining costs, e.g., public costs (interruption in works, childcare), social costs (disability pension), costs of restoration of health, and damages paid out.

Data on the manner of treatment (procedures and diagnoses, time needed for hospitalisation) of all analysed cases were then compared with the financing conditions made available by the National Health Fund’s president, the “NFZ president’s regulation” creating the conditions of establishing and performing contracts for hospital treatment, outpatient specialist care, hospital emergency department/admissions desk services, and therapeutic rehabilitation [20]. This listing of the most important treatment parameters has enabled the assigning of every case to a specific settlement version (the DRG group) with the National Health Fund. The payer, depending on patient’s age, diagnoses, performed procedures, and the manner of treatment, as well as the duration of the hospitalisation, specifies the amount of income for DRG groups, which the entity providing the medical service receives for performing it. The tariffs in force during a given year for the aforementioned groups are described in attachments to the aforementioned NFZ president’s regulations.

Therefore, the examined cases were grouped into appropriate categories depending on the method of treatment and on the actions performed for the patient (conservative treatment, surgical treatment), divided by hospital wards, as well as the manner of admission (emergency or planned treatment). For every analysed case, information is provided about the original stay (primary treatment) related to the main cause for hospital admission, e.g., diagnosing and treatment of emergency events (including heart failure, strokes, and injuries), or planned surgeries (arthroplasty, cataract, or bariatric surgery) during which the adverse event has occurred (according to Polish terminology: medical error, medical event, or hospital infection, etc.). Assigning every patient to a Diagnostic-Related Group (DRG) group enabled the indication of the costs of primary treatment which constitute income for an entity providing medical services, but at the same time a burden for the healthcare system in Poland. These are, however, expenditures which may not be avoided (the entity is required to provide the services in accordance with the contract signed with the payer) regardless of the circumstances, type, and complication of the case. Of course, the fact of occurrence of an adverse event in most cases will result in the generation of additional costs (medication, diagnostics, and repeated procedures) already during the primary treatment; however, due to the restrictions in access to such detailed data, this study did not manage to separate the “above-standard” costs from ordinary costs.

A similar assignment was made for subsequent stays, during which the main reason was the need to treat the patient in relation to the occurrence of an adverse event. Every repeated hospitalisation was assigned to an appropriate DRG group (surgical or conservative), depending on the activities and procedures performed for the patient. The manner of proceeding in many cases has resulted from the type and character of the adverse event, i.e., hospital infection, diagnostic error, or therapy error. In some cases, the treatment was finished (the patient did not feel any further negative consequences of the occurring situation), in other cases, the patients have suffered harm to their health and will never return to full health/physical condition. Therefore, the costs related to further treatment (e.g., rehabilitation, follow-up in specialist clinics) of such patients are not small at all. These include not only the expenses resulting directly from further surgeries/treatments or diagnostics, but also damages and compensation for the harmed patients and their families.

The aforementioned income for the entity is a burden and financial “loss” for the healthcare system, which instead of investing into new technologies must provide funds for the treatment of complications and consequences of adverse events. Without them, subsequent stays would not be necessary at all, and thus additional costs would not be generated in the system. The authors of the study have thus assumed all the elements relating to the treatment of the consequences of the recorder events are an avoidable expense, which could have been eliminated virtually entirely.

All the collected information was entered into an Excel spreadsheet. The statistical analysis necessary for the purpose of this publication was performed using the functions available within that software.

## 3. Results

**Figure 2:**
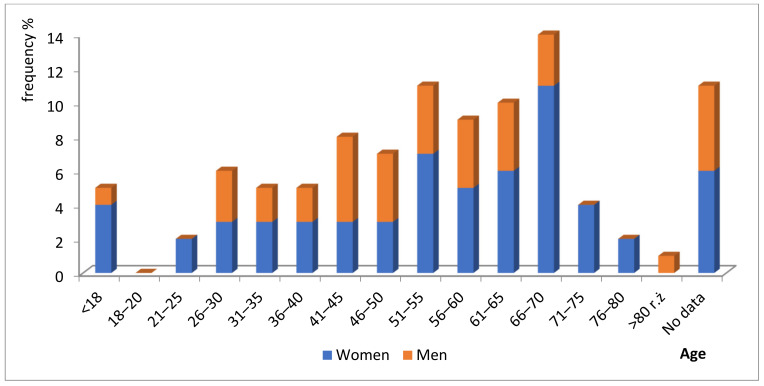
Age categories of patients divided by sex (%).

The statistical analysis of the examined cases enabled establishing that in 62% they concerned women. Only 38% were events which applied to men. Age categories of patients divided by sex were presented in Figure 2. Unfortunately, in 11% of cases, there was no information on this subject. In accordance with the “Safe Hospital Safe Patient” report by the Centre for Monitoring of Quality in Healthcare of 2014 [21], a hypothesis was presented that women care for their health more than men. That is why they more frequently receive medical services, which increases the risk of the occurrence of an adverse event during treatment. Additionally, women are characterised by a lower tolerance for treatment failure, which results in a higher share of women among patients seeking compensation through court proceedings. It may be simultaneously conjectured (although there was no such analysis conducted in this study) that women, being better educated, have more knowledge of patient rights, and also of the obligations of medical entities. Thus, they may have a higher awareness of the possible compensation/damages for incorrect treatment and a belief that they are entitled to it.

**Figure 3:**
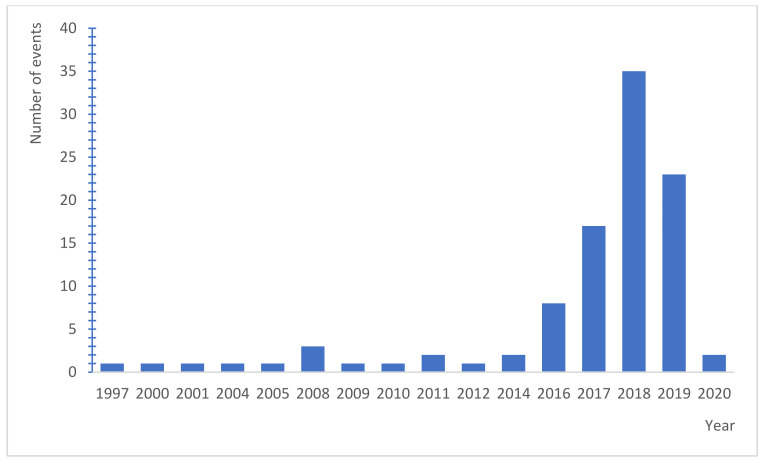
The number of events reported in individual years.

At the same time, the number of assessed claims was analysed for the year during which the adverse event occurred (Figure 3). It can be clearly seen that the highest number of cases concerned events which occurred in the last few years, that is 2018 (35%), 2019 (23%), and 2017 (17%). Sporadically, cases have concerned claims from much earlier (even from 1997). This is probably related to the growing awareness of patients and their families (knowledge of patient rights, issues of quality, and safety of care), as well as with the operation of province commissions for adverse events since 2012 (which in many cases enables assessing whether an adverse event has occurred or not more rapidly than in the courts). It should be kept in mind that the application has to be filed within a time limit of 1 year since the day on which the applicant has learned about the infection, bodily injury or disorder of health, or the patient’s death has occurred. This time limit may also not be longer than 3 years from the day on which the event has occurred. This type of information indicates clearly that over the last few years, a clear upwards trend can be noticed. The consequences of the higher number of proceedings for these kinds of crimes include, among others, the creation of special departments in regional prosecutor’s offices (since 2016) and the taking over of some of these cases by the offices of the district prosecutors. With these actions, the quality of procedures concerning medical errors has improved significantly. It may be thus expected that both the number and the value of awarded damages/compensation will be higher. Therefore, the burden on the healthcare system and on the entities which provide medical services will keep increasing.

It should be noted that the decrease in the number of claims in 2020 visible in the study does not only result in the delay in them being filed (up to 3 years form the event), but mainly from the introduction of the state of pandemic emergency within Poland (the Act of 2 March 2020 on special solutions related to preventing, counteracting, and combating COVID-19, other infectious diseases and emergencies caused by them). In accordance with the issued recommendations and guidelines, it was also necessary to limit the number of hospitalised patients, and also to transform wards or even entire hospitals into entities which provide services only to COVID-19 patients. Therefore, in most facilities, patients were admitted only in an emergency (risk to health/life), and the planned services or higher-risk surgeries were cancelled until further notice. Additionally, the feeling of risk resulting from the potential of death caused by a COVID-19 infection has resulted in a decrease in the reportability of patients to hospitals and in the use of medical care from the previous level. Thus, there is a significantly lower number of hospitalisations, and thus of adverse events reported in 2020 and claims resulting from them [22].

**Figure 4:**
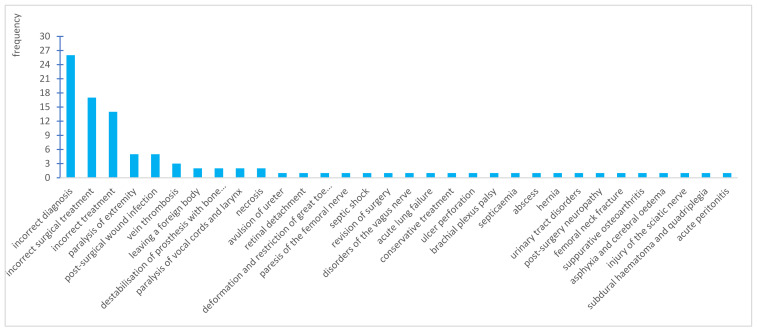
Type and amount of adverse events which form the basis for the claim.

Data concerning the type of adverse events to which the compensation claims applied were also analysed. Due to the diversity, they were grouped into 32 more general categories (Figure 4). The most frequent events included those related to incorrect diagnosis (the lack of correct diagnosis), which resulted in appropriate activities not being undertaken and a lack of appropriate treatment, e.g., lack of diagnosis of cancer, myocardial infarction, appendicitis, or fracture (26%). The next one was incorrect surgical treatment (17%)—the consequence of which was most frequently a need for repeated surgery and an incorrect conservative treatment of injuries (14%).

The aforementioned events most frequently resulted from an incorrect interpretation of symptoms, not using the available diagnostic capabilities (laboratory tests and imaging), a lack of appropriate competences, unjustified delays in commencing therapy or performing surgery (omission), incorrect pharmacological treatment, and organisational weaknesses. Similar results are presented in global publications [19,23,24,25].

**Figure 5:**
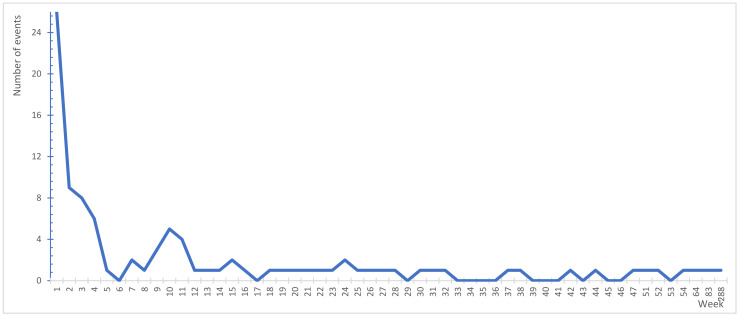
The time between the hospitalisation/stay resulting from the primary disease and the “secondary harm” for analysed cases (weeks).

The next step was an assessment of examined cases for the interval between hospitalisation/stay as a result of the primary disease, and the commencement of the treatment of the consequences of an adverse event—the “secondary harm” (Figure 5). For all analysed cases, the average time was 109.79 days (10 weeks), the median was 37.5 days (6 weeks), and the mode was 1 day (8%). At the same time, the maximum time amounted to 2010 days (288 weeks) and concerned a patient for whom it was necessary to perform a heart transplant surgery due to the lack of correct diagnosis and the lack of implementation of correct treatment. The patient has also a permanently issued disability certificate. It should be noted here that a disability certificate was issued for 18% of cases, in 6% of cases a consequence of the event was the death of the injured party, and for 5% of cases the treatment was not finished until today.

Then, in accordance with the assumptions of the study and based on directories which indicate the amount of financing of services by the National Health Fund, every case was assigned to the appropriate settlement group. This type of activity required the knowledge of the principles of coding and reporting of medical services to the NFZ payer. Assigning was performed both for the primary services (Table 1) and for subsequent services which were the result of the event (Table 2). This enabled the establishing of the probable costs which were borne by the healthcare system for the treatment of patients for whom the adverse event has occurred. It can be clearly seen that most of the events occurred as part of a stay at the admissions desk/hospital emergency department, and the treatment of the consequences of these events included hospitalisation at a surgical department (surgical treatment was marked with “*”). The occurrence of adverse events has thus implied the need for further, sometimes very long and very expensive treatment. After all, should the aforementioned situations not occur, the hospital stays and consultations would be unnecessary. Additionally, the funds expended on them could have been spent on other patients and procedures. For most cases, a confirmation of the fact of occurrence of an event was practically immediate, which enabled the implementation of immediate treatment, thus reducing future costs. For cases which were identified at a later time, the expenditures for the “restoration” of health were greater, if any actions could be undertaken at all. This study also does not raise the problem of costs generated for extended stays or additional diagnostics performed during the hospitalisation with the adverse event already. It is only restricted to the expenditures related to subsequent visits and the further use of services financed from public funds.

The financial data presented in the study are based on the principles of settlements made between the medical entities and the payer (NFZ) and the diagnosis-related group’s (DRG) price lists. We do not possess more precise information related to the detailed costs of treatment and their division depending on the procedure or the type of adverse event. At the same time, a relatively small (only 100 cases) and highly heterogeneous studied group did not enable the use of advanced methods (e.g., data mining, “Black Point Method for Adverse Events”), which would explain in a different manner the relationship between the expenditures of the system and the occurrence of adverse events.

It should be noted that the calculation of the aforementioned costs was based solely on data and information available to the authors on the day the materials were analysed. In cases when the treatment has not finished, only the already known expenses have been taken into account. It may impact the reliability of the obtained results and constitute a limitation of this study in relation to an underestimation of the actual costs of further care and treatment.

**Figure 6:**
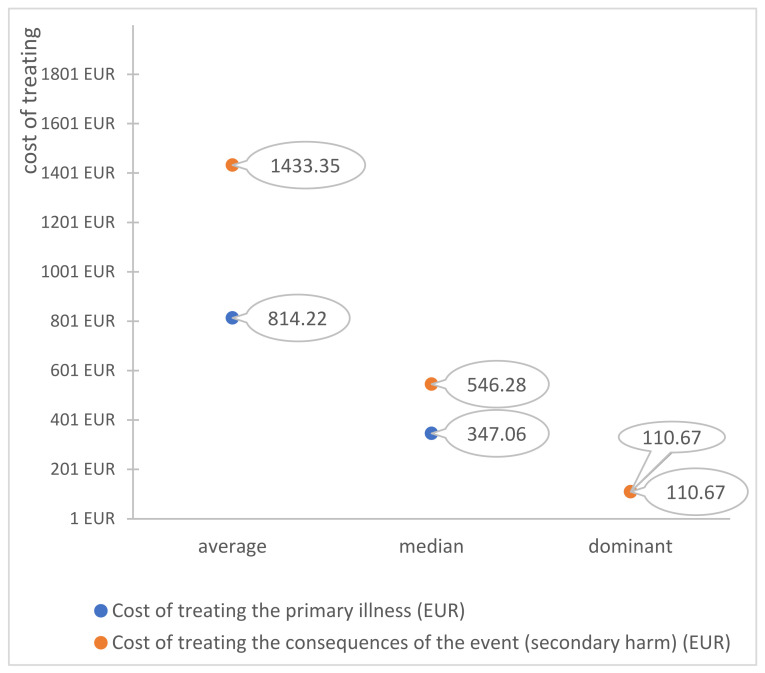
Cost of treating the primary illness and the consequences of the event (EUR).

The average, mode, and median of the costs of treatment, both primary and secondary, are presented in Figure 6. It can be clearly seen that the average cost of treatment of the consequences of adverse events are almost twice as high compared to the primary disease. The medical entities thus spend more funds on “restorative” actions, which constitute an attempt to protect against the negative consequences of wrong decisions or actions, than on the primary treatment. The presented amounts are also a burden for the entire healthcare system, which instead of focusing on appropriate treatment must finance additional corrective services (e.g., repeated surgeries, rehabilitation) not related to the primary illness.

All the costs calculated above related to the treatment of patients as a result of their claims should be also increased by additional costs related to the expenditure for commuting, treatment, or rehabilitation. In our study, it was an average amount of EUR 385.9 (PLN 1782.00).

## 4. Discussion

The Supreme Audit Office (NIK), based on information provided by the Patient’s Rights Ombudsman, has indicated that in the years 2012–2017, the 16 provincial committees adjudicating on medical events in Poland have received 5604 motions for establishing a medical event [26]. The committees have issued within this time a total of 1133 judgements establishing a medical event and 2111 judgements on the lack of such an event. The NIK also specifies that the 16 committees have received 1468 motions for the reconsideration of a case, and a total of 380 proceedings were discontinued.

At the same time, data from the Ministry of Justice on proceedings for damages handled by common courts since 2011 and damages and claims finally awarded within these courts for harm caused by medical entities [8] are presented in Table 3. The lists indicate that there is not a significant difference in the number of new court proceedings (lack of a growth trend). At the same time, the fact of an extension of the duration of proceedings (according to the CEPEJ methodology) is clearly visible [26], which translates to an increase in the number of cases that are not finished and carry over to subsequent years.

The Ministry of Justice also provides annual data concerning the number of cases and persons who were awarded damages/compensation, and their total amount. Similarly, as in our analysis, the year with the largest number of claims is 2018. In this year, the highest value of awarded compensation was also noted (Table 4).

In 2018, the Organisation for Economic Co-operation and Development (OECD) published a report called “The Economics of Patient Safety: Strengthening a Value-based Approach to Reducing Patient Harm at National Level” [27]. It unequivocally indicates that harm related directly to the provision of health services—in addition to the burden of morbidity, mortality, and disability—also generates high financial costs and burdens to every healthcare system in the world. At the same time, the type and severity of the adverse event have a significant impact on the costs borne by healthcare entities. These expenses are mainly related to additional diagnostics, doctor’s consultations, treatment, a longer hospital stay, as well as court proceedings and damages. The presented report also contains an analysis of the costs of adverse events in selected countries and their impact on healthcare resources (Table 5). Its authors report that the financial burden of all categories of adverse events which occur in hospitals is within 0.2–16.5% of the expenditure of public hospitals [28].

When analysing the available publications, it should be noted that adverse event claims focus mainly on surgical activities. According to reports by M. Bolcato, 11 out of 16 cases (69%) from the analysis of judicial and medical disputes belonged to this area. This only confirms the opinion that it is an area of high risk in healthcare. Simultaneously, no specific type of event repeats frequently enough to enable hypothesising that significant critical problems are focused on a single action in a hospital context [2]. Similar conclusions may be drawn from the analysis of our cases.

At the same time, the most frequent indirect causes of medical events indicated by doctors include an insufficient number of staff compared to the number of cases, excessive professional duties, the insufficient experience of medical personnel, and consultations conducted by doctors without specialisations [31]. Additionally, the available publications report that even up to 90% of all events occur on weekends and on holidays, which is closely connected to worse access to diagnostic and imaging examinations on these days, inadequate equipment and medical instrumentation, staffing irregularities at the level of the admissions desk or hospital emergency department that burden doctors with multiple simultaneous duties, and a lack of information flow between various professional groups and organisational units [32].

All the information presented and collected above necessitate the authors of this study to point out a significant problem of adverse events and their effects. Their consequences include not only health-related harm for the patient, but also long-term social, familial, or professional results. Very frequently when assessing, e.g., the costs of treatment of consequences of events, we forget about the “secondary harm”. According to the authors, “secondary harm” is a wider concept than an adverse event, since it contains its component, and moreover, it conveys not only medical and non-medical consequences for the person subjected to it and for the patient’s family, but also financial, legal, familial, social, and professional consequences. By presenting the occurrence of secondary harm on the time axis, it occurs on the day when the healthcare system allowed the deterioration of the patient’s health by inappropriate action or omission.

The authors of the article are also of an opinion that the insurance societies which participate in the process during loss adjustment should ensure the appropriate conduct of loss adjustment proceedings and the payment of benefits related to the patient’s claims or patient’s family’s claims (in case of the patient’s death) in order to redress the damage. The obligations of the insurance society should not be restricted only to the payment of benefits, but should also include processes which support medical facilities in the improvement of the quality of performed medical services and present them with reports which demonstrate the effects of the harm and good practices which result from problems concerning events in other medical facilities. This should result in education through the implementation of courses and workshops for the facility’s managers and personnel.

Additionally, the authors propose covering the parties injured by an adverse event (subjected to “secondary harm”) with a unique, innovative programme of post-accident health care called “Health Reconstruction”, resulting in the patient’s return to independence. This programme, in addition to a special “health audit” and organisation of a Health Reconstruction process assumes providing the patients with comprehensive medical care, rehabilitation, care services, and psychological support, which will enable a rapid return to health, minimising the effects of the adverse event. Being covered by the programme should be possible right after the event (according to the authors’ practice, it is necessary and essential, since only intensive medical and psychological help and support may demonstrate an appropriate direction which minimises the effects of health loss). We are aware of the fact of how important it is to immediately provide appropriate medical help, which is why support provided to the patient by specialists, medical consultants, and lawyers should occur right after the accident. The Health Reconstruction programme, as an answer to the harm which has occurred, would have as its aim the protection of health, and it should be financed from the funds handed over by the insurer or entity responsible for causing the harm (this may occur should the insurance society exceed the guaranteed sum resulting from the insurance policy, and benefits such as damages, compensation, and disability pension should be paid directly by the perpetrator of the event). The scope of the programme should be established based on an agreement regarding a report from a health audit, which would include the necessary services. What is most important is that in addition to the medical activities, the patient is supported in a return to normal life. These actions would include an adaptation to a new profession and other necessary types of support used to ensure that the patient is as able-bodied as possible. It should be also noted that the redress of damage also applies to the family of the injured person (which has died as a result of the adverse event), consisting of psychological and therapeutic aid, help in the organisation of a funeral, or the payment of benefits, that is damages for bereavement, appropriate compensation for the deterioration of material standing, and compensation related to the costs of transport, burial or funeral banquet, and an appropriate pension.

Being aware of the lack of possibility of the complete elimination of adverse events from the healthcare system, as well as the estimated scale of the phenomenon, we are convinced that these types of solutions and practices would enable a reduction in the negative consequences of their occurrence and the generated costs. In the situation of the occurrence of a problem, leaving the patient alone to themselves serves nothing. We think that every injured person should be provided with special care and supervision, a support which will enable implementing, as rapidly as possible, corrective and preventive actions before far reaching consequences.

## 5. Conclusions

All the conducted analyses and conclusions drawn from them should serve the improvement of patient safety. They also form an initial point for establishing recommendations and advice for the improvement of safety and quality of medical services and reduction of healthcare-related costs [33,34,35]. The main conclusions include:

1. Establishing the causes of adverse events enables the implementation of corrective (preventive) action, which should safeguard against the repeated occurrence of similar situations in the future and against negative repercussions;
2. The treatment of consequences of adverse events places a significant financial burden on the healthcare system;
3. The consequences of adverse events include not only health-related harm for the patient, but also long-term social, familial, or professional results;
4. The authors propose covering the parties injured by an adverse event (subjected to “secondary harm”) with a unique, innovative programme of post-accident health care, “Health Reconstruction”, with the aim being a return to independence.

## Figures and Tables

**Table 1:** Type of provided services, their number, and costs of treatment of primary disease in accordance with the directories by the National Health Fund President.

DRG Group/Settlement Service Code	DRG Group/Settlement Service Name	Estimated Value of Payment Acc. to NFZ Directories (EUR)	Number of Cases
5.53.01.00050	Treatment in the ICU for adults—assessment acc. to TISS-28 scale	11,815.2	1
A35D	Degenerative diseases of the CNS > 3 days	720.6	1
A45	Cerebrovascular diseases—conservative treatment	685.7	1
A57	Inflammatory diseases of the nervous system	894.8	1
A76	Head trauma with significant brain damage—treated conservatively	348.6	1
A77	Head injuries without significant brain damage—treated conservatively	185.9	1
A87	Other nervous system diseases	346.4	2
B03	Treatment with verteporfin with the use of photodynamic therapy *	1580.6	1
B17/B23	Vitrectomy procedures, including multiple procedures */Medium procedures on the lens *	1408.0	1
B19G	Cataract removal—Category II *	499.6	1
C12	Major procedures on oral cavity, pharynx, and larynx *	547.1	1
C14	Medium procedures on oral cavity, pharynx, and larynx *	216.6	1
C57	Other diseases of throat, ears, and nose	255.7	1
D18	Atypical viral pneumonia	836.8	1
E27	Coronary catheterisation and other invasive procedures *	413.5	1
E57	Ischemic heart diseases > 17 years of age or < 70 years of age without complications	431.1	1
E73	Heart valve diseases > 17 years of age	665.8	1
F14	Surgical treatment of obesity *	2446.0	1
F16E	Stomach and duodenum diseases > 65 years of age	480.5	1
F32	Major and endoscopic procedures on the large intestine *	1265.2	1
F43E	Medium and endoscopic treatments of the abdominal cavity > 65 years of age *	650.7	2
F43F	Medium and endoscopic treatments of the abdominal cavity < 66 years of age *	546.3	1
G21	Comprehensive procedures on bile ducts *	2972.5	1
G25F	Cholecystectomy < 66 years of age	732.2	1
H13	Primary total hip arthroplasty *	2320.9	2
H23	Diagnostic and therapeutic arthroscopy *	330.6	1
H32	Major procedures on the lower limb and pelvis *	1034.3	1
H33	Medium procedures on lower limb *	290.5	2
H43	Medium procedures on upper limb *	290.5	1
H51	Comprehensive spinal correction surgeries *	5857.3	1
H52	Spinal surgeries with the use of implants *	3077.5	1
H53	Spinal surgeries without the use of implants *	2091.8	1
H62E	Breaks or dislocations of the pelvis or lower limb > 65 years old *	1786.7	1
H62F	Breaks or dislocations of the pelvis or lower limb < 66 years old *	1144.3	3
H63	Breaks of dislocations of the upper limb *	825.6	1
H64	Minor breaks or dislocations *	363.6	1
H67	Functional treatment of breaks of long bones, joints, pelvis, or spine	1023.6	1
H84	Minor procedures within the musculoskeletal system or soft tissues *	347.7	1
AD/ED	AD/ER stay (averaged cost per patient acc. to daily lump sum payment)	110.7	37
K01	Radical surgeries in endocrine gland cancer *	3184.8	2
K02	Surgery of nodular goitre with complications *	1499.1	2
K59	Other diseases of the endocrine system	421.7	1
L06	Medium endoscopic kidney procedures *	464.8	1
L82	Acute kidney failure	918.1	1
M05	Urinary incontinence surgeries *	747.2	1
M14	Medium procedures on the upper genital tract *	546.3	2
M26	Conservative treatment on the upper genital tract	178.8	1
N03	Pregnancy or foetus pathology with birth > 5 days *	625.8	1
N25	Neonate requiring increased surveillance	639.1	1
Q23	Varicose vein surgeries with saphenectomy	430.0	1
Q41	Endovascular procedures—1st group *	1115.8	1
T06	Other surgeries for multiple injuries *	3742.3	1
T07	Conservative treatment of injuries	190.4	3

* surgical treatment.

**Table 2:** Type of provided services, their number, and costs of treatment of the consequences of event (secondary harm) in accordance with the directories by the National Health Fund President.

DRG Group/Settlement Service Code	DRG Group/Settlement Service Name	Estimated Value of Payment Acc. to NFZ Directories (EUR)	Number of Cases
5.11.01.0000073	Individual work with the patient (e.g., passive exercises, active/passive exercises, neurophysiological method exercises, neuromuscular re-education methods, special exercises, and mobilisations and manipulations)—no less than 30 min *	126.4	2
5.53.01.00050	Treatment in the ICU for adults—assessment acc. to TISS-28 scale	3475.1	1
5.53.01.00050	Treatment in the ICU for adults—assessment acc. to TISS-28 scale	9730.1	1
A31	Peripheral nerve diseases	335.2	1
A76	Head trauma with significant brain damage—treated conservatively	335.2	3
A87	Other nervous system diseases	360.2	5
B17	Vitrectomy procedures, including multiple procedures *	1408.0	1
B19G	Cataract removal—Category II *	499.6	1
C11	Comprehensive procedures on oral cavity, pharynx, and larynx *	2507.6	2
C57	Other diseases of throat, ears, and nose	255.7	2
D05	Bronchoscopy *	156.4	1
D52	Respiratory failure	557.9	1
E11	ACS—two-stage invasive treatment > 3 days *	3283.0	1
E27	Coronary catheterisation and other invasive procedures *	413.5	1
E43	Ablation of arrhythmias *	3486.5	1
E53G	Circulatory failure	742.3	1
E59	Sudden cardiac arrest	726.4	1
F14	Surgical treatment of obesity *	424.6	1
F16F	Stomach and duodenum diseases < 66 years of age	430.0	1
F31	Complex treatments of the large intestine *	2906.6	1
F83	Appendectomy *	546.3	1
F43E	Medium and endoscopic treatments of the abdominal cavity > 65 years of age *	638.3	1
F43F	Medium and endoscopic treatments of the abdominal cavity < 66 years of age *	546.3	2
F44	Diagnostic and treatment abdominal cavity procedures *	335.2	1
F58F	Inflammatory diseases of the intestines > 66 years of age	929.7	1
F82	Appendectomy with complications *	802.0	1
F83	Appendectomy *	546.3	2
G01	Extensive procedures of the liver *	3265.6	1
G18	Chronic liver disease without complications	478.7	1
G21	Comprehensive procedures on bile ducts *	3091.3	1
H11	Resection surgeries of cancerous and tumour-like lesions with arthroplasty or surgical revision with post-resection prostheses *	2859.0	1
H13	Primary total hip arthroplasty *	2320.9	3
H17	Primary total hip revision arthroplasty *	3007.1	1
H23	Diagnostic and therapeutic arthroscopy *	337.0	1
H33	Medium procedures on lower limb *	285.0	3
H43	Medium procedures on upper limb *	290.5	1
H51	Comprehensive spinal correction surgeries *	5857.3	2
H53	Spinal surgeries without the use of implants *	2091.8	1
H62F	Breaks or dislocations of the pelvis or lower limb < 66 years old *	1144.3	2
H63	Breaks of dislocations of the upper limb *	825.6	4
H64	Minor breaks or dislocations *	363.6	1
H72	Extensive and major amputations *	1329.8	1
H83	Medium procedures on soft tissue *	424.6	1
H84	Minor procedures within the musculoskeletal system or soft tissues *	347.7	1
H90	Arthritis and connective tissue inflammation diseases which require intensive therapy > 10 days	1394.7	1
AD/ED	Stay at the admissions desk/emergency department (averaged cost per patient acc. to daily lump sum payment)	110.7	7
J33	Medium dermal procedures *	391.1	1
J34	Surgery of the trophic lesions of the foot *	927.5	1
J46	Major skin infections	464.8	1
L00	Nephrectomy and other major open kidney surgery *	1766.5	1
L104	Other urogenital tract procedures *	89.0	1
M13	Major procedures on the upper genital tract *	790.2	1
M16	Miscarriage or risk of miscarriage, termination of foetal death *	325.4	1
N25	Neonate requiring increased surveillance	639.1	1
W12 advice	W12 Type 2 specialist service	14.0	1
W13 advice	W13 Type 3 specialist service	26.0	1
AMD programme	Ambulatory admission of the patient combined with anti-VEGF intravitreal injection in a drug programme	81.4	1
heart transplant	Version 1—heart transplant	31,216.2	1
PZN01	Comprehensive surgeries of neonates and infant *	4839.3	1
Q12	Procedures on lower limb arteries *	1708.5	1
Q14	Stenting and reconstructing of extracranial and upper limb vessels *	1732.5	1
Q19	Medium procedures on the lymphatic system *	464.8	1
Q66	Vascular diseases	609.2	2
ROO03	General rehabilitation after surgery	1087.4	4
S52	Immunodeficiencies other than HIV/AIDS	569.5	1
T06	Other surgeries for multiple injuries *	3742.3	1
T07	Conservative treatment of injuries	190.4	4

* surgical treatment.

**Table 3:** Record of compensation cases for harm caused by the healthcare system in the years 2011–2020—own work.

Year	Received	Handled	Including							Remaining for the Next Period	Indicator of Duration of Proceedings (Acc. to CEPEJ Methodology) in Days
			Allowed Fully or Portali	Discontinued		Dismissed	Return of Claim/Motion	Rejection of Claim/Motion	Other Handling		
				Total	Including a Settlement Was Made						
**Regional Courts 1st instance**											
2011	613	550	140	61	18	195	42	10	102	1 208	801,7
2012	746	537	148	33	5	187	63	13	93	1 417	963,1
2013	773	612	153	56	14	234	76	10	83	1 575	939,3
2014	725	700	199	49	15	235	72	9	136	1 600	834,3
2015	753	637	193	52	11	217	72	10	93	1 716	983,3
2016	604	629	190	49	23	233	79	14	64	1 691	981,3
2017	683	609	172	50	12	235	66	5	81	1 765	1057,8
2018	675	635	171	50	14	250	58	5	101	1 805	1037,5
2019	692	647	176	64	29	207	81	8	111	1 851	1044,2
2020	653	606	153	60	22	175	73	8	137	1 898	1143,2
**Regional Courts 2nd instance**											
2011	52	44	2	1	0	49	10	2	2	16	132,7
2012	73	66	3	0	0	36	16	9	2	22	121,7
2013	38	46	2	1	0	31	10	1	1	14	111,1
2014	51	46	2	0	0	26	18	0	0	19	150,8
2015	45	51	2	0	0	35	13	0	1	13	93,0
2016	47	40	3	1	0	24	12	0	0	20	182,5
2017	42	44	2	0	0	31	11	0	0	18	149,3
2018	38	43	3	0	0	26	12	0	0	13	110,3
2019	41	26	0	0	0	20	6	0	0	28	393,1
2020	32	33	1	0	0	27	5	0	0	27	298,6
**Regional Courts**											
2011	249	219	51	17	4	65	42	5	39	304	506,7
2012	225	264	52	30	3	66	50	6	60	265	366,4
2013	217	187	37	25	3	61	21	3	40	295	575,8
2014	196	191	41	22	7	48	30	2	48	300	573,3
2015	194	194	37	29	10	52	23	3	50	300	564,4
2016	150	156	36	21	4	49	18	1	31	294	687,9
2017	164	157	44	13	5	54	19	1	26	301	699,8
2018	182	173	27	14	2	49	28	8	47	310	654,0
2019	138	146	47	11	1	46	11	1	30	302	755,0
2020	141	156	36	16	4	34	12	1	49	286	669,2
**Courts of Appeals**											
2011	239	225	16	1	0	127	71	7	2	69	111,9
2012	253	234	18	2	0	135	59	8	12	88	137,3
2013	264	215	16	2	0	109	72	9	7	137	232,6
2014	270	252	17	3	1	164	59	7	2	155	224,5
2015	280	254	10	2	0	148	86	6	2	181	260,1
2016	260	228	13	0	0	144	62	4	4	213	341,0
2017	267	279	8	2	0	147	91	2	38	201	263,0
2018	266	237	11	4	0	126	88	1	6	230	354,2
2019	243	226	13	3	0	138	67	2	2	247	398,9
2020	219	219	11	4	0	117	74	10	3	247	411,7
**Total**											
2011	1 153	1 038	209	80	22	436	165	24	145	1 597	1 553
2012	1 297	1 101	221	65	8	424	188	36	167	1 792	1 588
2013	1 292	1 060	208	84	17	435	179	23	131	2 021	1 859
2014	1 242	1 189	259	74	23	473	179	18	186	2 074	1 783
2015	1 272	1 136	242	83	21	452	194	19	146	2 210	1 901
2016	1 061	1 053	242	71	27	450	171	19	99	2 218	2 193
2017	1 156	1 089	226	65	17	467	187	8	145	2 285	2 170
2018	1 161	1 088	212	68	16	451	186	14	154	2 358	2 156
2019	1 114	1 045	236	78	30	411	165	11	143	2 428	2 591
2020	1 045	1 014	201	80	26	353	164	19	189	2 458	2 523

**Table 4:** Finally awarded compensation and damages for harm caused by the healthcare system in the years 2011–2020—own work.

Year	Number			Total Value of Awarded Damages (EUR)	Total Value of Compensation (EUR)
	Cases	Persons Awarded			
		Damages	Compensation		
**Regional Courst**					
2011	27	0	0	125 551,81	38 321,45
2012	22	0	0	79 773,34	71 813,65
2013	14	16	0	83 725,80	0,00
2014	19	20	8	71 567,37	15 040,00
2015	55	48	10	165 812,48	21 881,79
2016	10	9	2	68 593,21	8 695,00
2017	7	5	3	4 939,14	9 870,00
2018	17	12	10	56 123,31	46 107,00
2019	11	7	5	28 628,23	21 737,50
2020	11	10	4	58 174,49	26 771,44
**Regional Courst of 1st instance**					
2011	41	0	0	550 114,56	903 629,29
2012	38	0	0	202 930,96	772 072,76
2013	30	16	30	81 694,70	747 799,14
2014	36	23	27	624 637,05	401 897,24
2015	42	25	36	210 955,27	741 558,01
2016	42	18	36	279 796,85	792 890,31
2017	27	18	27	266 609,34	748 594,85
2018	57	34	57	303 409,22	1 863 722,96
2019	45	19	40	361 517,42	1 139 027,85
2020	25	13	15	596 280,54	795 827,74
**Regional Courst of 2nd instance**					
2011	7	0	0	7 233,30	104 575,00
2012	5	0	0	19 339,80	36 425,00
2013	10	6	4	54 990,92	13 195,25
2014	17	11	4	52 578,90	24 675,00
2015	14	9	6	20 633,00	15 627,50
2016	6	3	3	22 325,00	7 755,00
2017	18	14	6	30 777,89	60 841,50
2018	20	16	7	36 077,67	38 923,05
2019	6	3	3	31 480,84	7 974,26
2020	9	2	7	1 024,84	36 589,50
**Courts of Appeals**					
2011	66	0	0	164 453,94	1 470 910,36
2012	69	0	0	202 562,01	2 011 043,76
2013	69	29	58	395 028,64	2 547 376,50
2014	56	23	64	264 106,49	1 600 884,63
2015	70	28	72	372 927,38	2 946 802,01
2016	74	22	72	381 148,30	2 994 464,71
2017	81	22	88	154 603,21	2 984 257,25
2018	85	28	90	377 514,99	2 803 470,60
2019	59	18	71	215 428,73	3 024 863,37
2020	62	17	76	167 026,96	2 866 823,75
**Total**					
2011	141	0	0	847 353,60	2 517 436,09
2012	134	0	0	504 606,10	2 891 355,17
2013	123	67	92	615 440,05	3 308 370,89
2014	128	77	103	1 012 889,81	2 042 496,86
2015	181	110	124	770 328,12	3 725 869,31
2016	132	52	113	751 863,36	3 803 805,02
2017	133	59	124	456 929,58	3 803 563,60
2018	179	90	164	773 125,18	4 752 223,61
2019	121	47	119	637 055,21	4 193 602,97
2020	107	42	102	822 506,82	3 726 012,42

**Table 5:** Costs of adverse events in hospitals [27,28,29,30].

Location, Temporal Scope	Amount	Share in Expenditures of Public Hospitals [%]
Ireland (2009)	EUR 194 million	4
Canada (2009–2010)	CAD 1.071 billion	4.2
Australia, without Victoria (2013)	AUD 634–896 million	12–16.5
Victoria, Australia (2009)	AUD 460 million	15.7
Europa (2016)	EUR 2.8–84.6 trillion	0.2–6
Netherlands (2009)	EUR 355 million	1.8
United States (2014)	Long-term care—2% of all MediCare expenditure is related to the treatment of adverse events	2
Denmark (2013)	EUR 3.1 billion	
Spanish (2013)	EUR 1.062 billion	1.5
England (2020–2021)	GBP 2.2 billion	1.5
Australia (2013)	AUS 1.2 billion	3.95

## Data Availability

The data presented in this study are available on request from the corresponding author.
